# Analysis of error type and frequency in apraxia of speech among Portuguese speakers

**DOI:** 10.1590/S1980-57642010DN40200004

**Published:** 2010

**Authors:** Maysa Luchesi Cera, Thaís Soares Cianciarullo Minett, Karin Zazo Ortiz

**Affiliations:** 1 Speech Therapist, Specialization in Human Communication Disorders at the Federal University of São Paulo (UNIFESP); Masters in Human Communication Disorders (UNIFESP), São Paulo SP, Brazil.; 2 Speech Therapist, PHD, Professor of the Department of Speech Therapy of the Federal University of São Paulo, São Paulo SP, Brazil.; 3 Neurologist, PHD, Professor of the Department of Preventive Medicine of the Federal University of São Paulo, São Paulo SP, Brazil.

**Keywords:** articulation disorders, apraxias, diagnosis, rehabilitation of speech and language disorders

## Abstract

Most studies characterizing errors in the speech of patients with apraxia involve English language.

**Objectives:**

To analyze the types and frequency of errors produced by patients with apraxia of speech whose mother tongue was Brazilian Portuguese.

**Methods:**

20 adults with apraxia of speech caused by stroke were assessed. The types of error committed by patients were analyzed both quantitatively and qualitatively, and frequencies compared.

**Results:**

We observed the presence of substitution, omission, trial-and-error, repetition, self-correction, anticipation, addition, reiteration and metathesis, in descending order of frequency, respectively. Omission type errors were one of the most commonly occurring whereas addition errors were infrequent. These findings differed to those reported in English speaking patients, probably owing to differences in the methodologies used for classifying error types; the inclusion of speakers with apraxia secondary to aphasia; and the difference in the structure of Portuguese language to English in terms of syllable onset complexity and effect on motor control.

**Conclusions:**

The frequency of omission and addition errors observed differed to the frequency reported for speakers of English.

## Abstract (pt)

Em geral, os estudos sobre os erros cometidos por falantes com apraxia de fala são internacionais.

**Objetivos:**

Analisar os tipos e as frequências dos erros presentes na fala de pacientes com apraxia de fala, falantes do português, falado no Brasil.

**Métodos:**

Participaram do estudo 20 adultos com apraxia de fala pós acidente vascular cerebral. Os tipos de erros cometidos pelos pacientes foram analisados quantitativa e qualitativamente e suas frequências foram comparadas.

**Resultados:**

Observamos a presença de substituição, omissão, ensaio articulatório, repetição, autocorreção, antecipação, adição, reiteração e metatese, respectivamente, de acordo com as maiores médias obtidas. O erro do tipo omissão foi um dos mais frequentes e o erro do tipo adição foi um dos menos frequentes. Estes resultados são diferentes de estudos com pacientes falantes do inglês e podem estar relacionados à diferença de metodologia para a classificação dos tipos de erros; à inclusão de pacientes com apraxia de fala associada à afasia; e à diferença de estrutura da língua Portuguesa em relação à Inglesa, que altera a complexidade da sílaba inicial e seu efeito no controle motor.

**Conclusões:**

A freqüência de ocorrência dos erros de omissão e adição diferiram de sujeitos falantes do inglês.

Apraxia of speech is an articulation disorder resulting from brain damage affecting the capacity to program the positioning of speech musculature and the sequencing of muscle movements for volitional production of phonemes.^1^ Many previous studies^1-17^ have described the manifestations of this disorder as well as the most frequent phonological errors. However, these reports typically involve international studies in English language speakers. A previous Brazilian study described those phonemes most frequently affected by substitution and omission errors committed by speakers of Portuguese with apraxia, and detected differences in comparison to international studies, although types of errors and their frequencies of occurrence were not reported.^3^

Therefore, the aim of the present study was to analyze the type and frequency of errors present in the speech of individuals with apraxia of speech whose mother tongue was Brazilian Portuguese.

## Methods

This study was approved by the Research Ethics Committee of the Federal University of São Paulo (UNIFESP) under protocol number 1105/07. All participants signed a free and informed consent form.

Participants were recruited from patients assessed at the Center for Speech and Hearing Investigation in Neuropsycholinguistics of Unifesp, who were diagnosed with apraxia of speech during 2007, according to the presence of the following types of error: metathesis, anticipation, reiteration, substitution, repetition, omission, addition, self-correction, trial-and-error, where these errors are typical of the oral production of apraxics.

For study inclusion, participants had to present a neurological diagnosis of a single lesion to the left-hemisphere and be native speakers of Brazilian Portuguese. The sample also included individuals with apraxia and associated aphasia since few patients present with apraxia of speech only.

Individuals with a marked expressive deficit, characterized by suppressed or severely reduced oral capacity; impaired auditory comprehension preventing task execution; clinical history or diagnosis of previous neurological conditions (such as epilepsy, head trauma with loss of consciousness of longer than 15 minutes), uncorrected hearing or visual disturbances, history of severe depression or psychiatric disorders, or use of psychotropic drugs, were excluded.

The final sample comprised 20 adults aged between 41 and 80 years (mean 58.8±10.4), with 11 men and 9 women. Three patients were diagnosed with hemorrhagic cerebral stroke while the remainder had suffered ischemic strokes. All patients with apraxia but one, were also aphasics. In terms of lesion site, six patients presented temporoparietal lesions, four fronto-temporal, three fronto-parietal, two parietal, two frontal, one temporal, one temporo-parieto-occipital and one parietal-occipital lesions.

Data was first gathered through anamneses (personal details and neurologic history). Speech assessment was carried out using the verbal praxic component of the protocol for evaluation of verbal and non-verbal apraxia,^18^ which entails tests of word and sentence repetition, automatic and spontaneous speech and oral reading aloud. The “Cookie Theft” test from the Boston Diagnostic Aphasia Examination was used to elicit spontaneous speech production.^19^

Patient speech was digitally recorded using a SONY MP3 player and concomitantly transcribed. The transcription of the speech, and data analysis were performed by the author of this study with the supervision of co-author (K.Z.O).

Initially, the presence of the following types of error was verified: metathesis, anticipation, reiteration, substitution, repetition, omission, addition, self-correction, trial-and-error, where these errors are typical of the oral production of speakers with apraxia. Substitutions, omissions, additions and repetitions are considered phonemic errors, where substitution occurs when a one phoneme is replaced by another, omission when one phoneme or syllable is dropped, addition when one phoneme or syllable is introduced to the word, and repetition where the sound, word, part of a word or utterance are produced more than once. Sequential errors were also analyzed, where anticipation is the early occurrence of a phoneme contained in the target word, reiteration is the repetition of a phoneme which has previously appeared in the target word, and metathesis is the sequential inversion of the phonemes within a word. Errors were categorized as being of the self-correction type when the patient produced the word or phrase incorrectly and spontaneously performed self-correction to then successfully produce the word or phrase. The trial-and-error error was registered when the participant sought the articulatory point of a phoneme or sequence of phonemes, in a bid to perform the correct movement, prior to initiating speech production.

With regard to other manifestations, hesitation is characterized by delay in initiating speech and undue prolonging of sounds.

All errors detected in patients’ speech were first compiled by quantity and type.

### Statistical analyses

Differences in the means of continuous measurements were tested by the Student’s t test for paired samples (t) and checked by the Wilcoxon’s test. As both tests yielded similar results in all cases, only the results of the parametric tests are presented. Multiple comparisons were undertaken and the p value was set at (p<0.006) according to Bonferroni correction. All tests were two-tailed. Ninety-five percent confidence intervals (95%CI) were calculated for differences between means. All analyses were performed using version 11.5.1of the SPSS (Statistical Package for the Social Sciences) statistical package for Windows.

## Results

Table 1 shows the descriptive analysis of the types of speech errors in patients with apraxia of speech.

**Table 1:** Descriptive analysis of types of speech error in patients with apraxia of speech.

Types of error	Mean	Standard deviation	Median	Minimum	Maximum
Substitution	12.9	10.4	11.5	1	49
Omission	12.5	9.7	11.0	0	46
Trial-and-error	10.0	6.7	9.5	1	22
Repetition	6.1	7.4	4.0	0	28
Self-correction	5.0	3.7	4.5	1	13
Anticipation	1.5	1.4	1.5	0	5
Addition	1.4	1.3	1.0	0	4
Reiteration	0.5	0.6	0.0	0	2
Metathesis	0.4	1.1	0.0	0	5

The means of different types of errors were compared to ascertain the most frequent error types (Table 2).

**Table 2:** Comparison of means for types of speech error committed by patients with apraxia of speech, according to student's t test for paired samples.

Comparison			Difference between means	95% CI (difference)			t(19)	P
Substitution	×	Omission	0.5	-5.6	to	6.5	0.2	0.878
Omission	×	Trial-and-error	2.5	-3.7	to	8.6	0.8	0.412
Trial-and-error	×	Repetition	4.0	0.8	to	7.1	2.7	0.016
Repetition	×	Self-correction	1.1	-2.2	to	4.3	0.7	0.510
Self-correction	×	Anticipation	3.6	1.7	to	5.4	3.9	0.001*
Anticipation	×	Addition	0.1	-0.7	to	0.9	0.3	0.804
Addition	×	Reiteration	0.9	0.2	to	1.6	2.6	0.018
Reiteration	×	Metathesis	0.1	-0.5	to	0.7	0.3	0.748

The results show that in terms of numbers of errors, the types of error substitution, omission, trial-and-error, repetition and self-correction were significantly more common than anticipation, addition, reiteration and metathesis errors.

**Figure 1:**
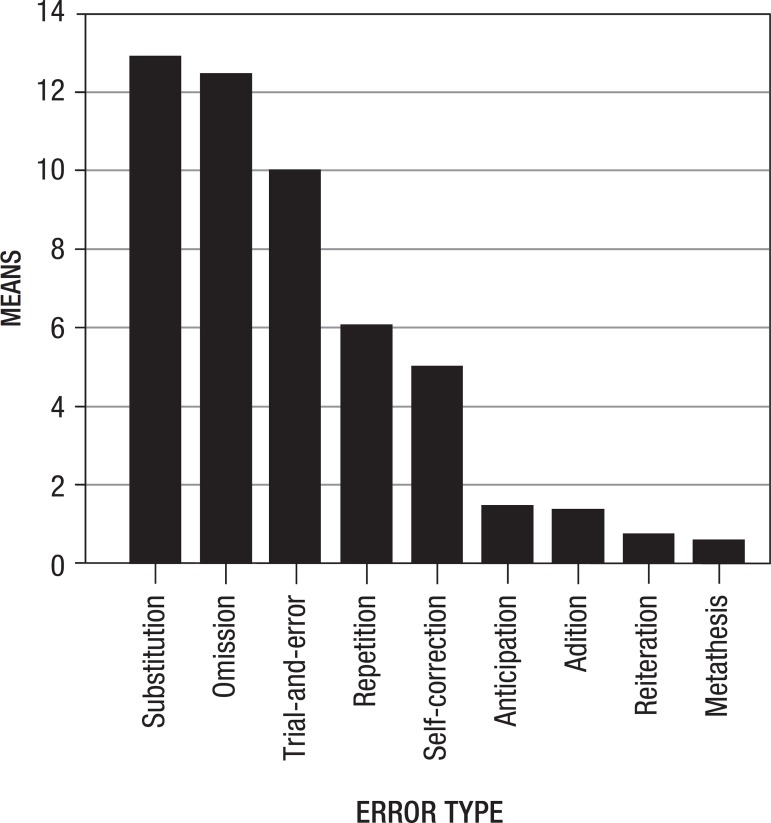
Distribution of error types as a function of mean frequency.

Figure 1 illustrates the relationships of means of speech error types.

Regarding other manifestations, comparison of means of hesitation and prolongation revealed no statistically significant differences (9.7±8.8versus 5.3±8.9, 95% CI= –1.5 to 10.3, t(19)=1.55, P=0.137).

**Figure 2:**
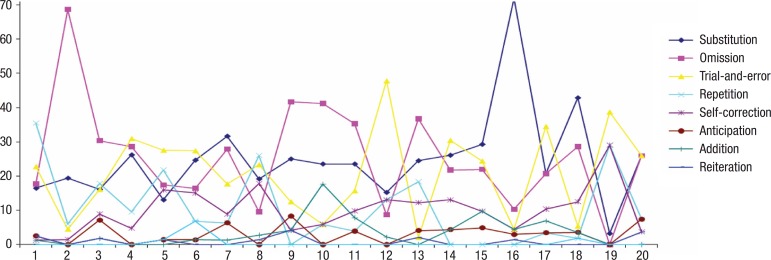
Frequency of error types in individual speakers.

Figure 2 shows the frequency of the types of errors by individual speakers.

## Discussion

Based on the error types analyzed and shown in Table 1, we noted the presence of substitution, omission, trial-and-error, repetition, self-correction, anticipation, addition, reiteration and metathesis, in descending order of frequency, respectively.

Numerous studies have also shown the substitution error type to be frequent in the speech of patients with apraxia.^4-6,8-9,14-15,20^ Mirroring the results found in speakers of other languages, we also found the substitution error type to be the most frequently occurring in Portuguese speakers. This error perhaps constitutes the most characteristic error of the apraxia of speech picture.

The omission type error had the second highest mean frequency in the present study, where this finding differed to the results of many previous studies.^4,8,15^ Johns and Darley (1970) found that the omission type error represented less than 1% of total errors^8^ whereas the study by Darley et al. (1975) showed that the most common errors in apraxia were: substitutions, additions, repetitions and phonemic prolongations.^4^ Peach and Tonkovich (2004) observed substitution errors, followed by addition, repetition, intrusion, omission and other error types.^15^ However, most of these studies involved English language speakers. Nevertheless, several other studies have also reported high omission occurrence.^20-21^ Odell et al. (1990) found a predominance of the distortion error type, followed by omission.^21^ The second highest mean found in their study also involved omission errors, although these authors had included the distortion error type in the data analysis. They also identified a discrepancy between their findings and those of other studies, ascribing this to differences in the methodologies employed by the different studies. In addition, they observed 14 types of distortion, the most common of which was prolongation, followed by devoicing. In our study for instance, prolongation was included in the analysis of other manifestations whereas devoicing was considered a substitution type error given that essentially one sound phoneme is being replaced by another.

Other authors have also considered distortion as a distinct error type after analyzing speech errors among this patient group.^8-10^ Johns and Darley (1970) defined distortion as the inaccurate production of a phoneme which is consequently rendered unrecognizable.^8^ These same authors compared the performance of apraxia and dysarthria and verified that apraxia presented only 10% distortion type errors while patients with dysarthria presented 65% of this error type. Although these authors found participants with apraxia to present more substitution and repetition errors, the inclusion of the distortion error type is incongruent with the scope of our study, since this type of error is not included in the analysis of speech samples of the participants. According to Joseph et al. (2006), apraxia of speech is characterized by the presence of distortion in consonants and vowels, sound substitution, addition, prolongation, trial-and-error and attempts at self-correction, slow rate of speech, prolongation and variation in vowel duration and, between words, syllable segregation and reduced phonetic accuracy at higher speech rates.^10^ In the present study, distortion was not included in the classification of error types, because no consensus has yet been reached in the literature on the classification of this error type.

Concerning trial-and-error, the literature indicates this is a characteristic of the symptom complex of patients with apraxia, although studies describing this manifestation have not examined it in the context of other error types.^7,10,16^ In our study, trial-and-error was the fourth most common type of error.

Some studies have addressed repetition type errors when describing speech manifestation of patients with apraxia,^4,5,7,8,10,15^ but did not compare them against the other error types.

Concerning self-correction, Wertz et al. (1984) reported that patients with apraxia of speech present attempts at self-correction.^16^ Wolk (1986) reported that successive attempts at self-correction tend to reveal refinement to closer reflect the target segment in terms of phoneme complexity (unmarked to marked).^17^ Liss (1998) found that speakers with apraxia of speech presented less evidence of efficiency in pre-articulatory monitoring, evidenced by a longer time interval between the interruption of the flow of speech upon recognizing an error and the commencement of revision, suggesting compromise in the ability to plan the revision prior to production.^13^ We hypothesize that participants may be demonstrating difficulty in articulatory motor planning, since when carrying out self-correction, they do not revise the inadequate motor planning prior to execution. This difficulty in planning and performing revision prior to production is evident from the occurrence of trial-and-error, in which the individual successively seeks the articulatory movement required.

Self-correction during the speech of these participants appears to involve the processing of feedback on the information. In studies examining visuomotor tracking abilities among individuals with apraxia of speech, Robin, Jacks, Hageman, Clark, Woodworth (2008) suggested that apraxia of speech results from a deficit in the feedforward motor control processes,^22^ conceptualized in the DIVA model of speech processing. When disturbances do occur, this system requires the control provided by feedback. Guenther et al. (2006), using the DIVA model of speech production, described the functioning of the feedback control subsystem in the event of errors.^23^ According to the authors, activation of the speech sound map cell corresponding to the sound in the model’s premotor cortex leads to readout of learned auditory and somatosensory targets for that sound, while error events are detected by the sensory cortex. These error signals are then mapped into appropriate corrective motor commands via learned projections for the sensory error cells of the motor cortex. With regard to the feedforward control subsystem, there are reports that during this early production, the system is “tuning itself up” by monitoring the motor commands generated by the feedback control system. The feedforward system improves over time, all but eliminating the need for feedback-based control except when external constraints are applied to the articulation, or auditory feedback is artificially disturbed. Once an appropriate feedforward command sequence has been learned for a speech sound, this sequence will successfully produce the sound with very little, if any, contribution from the feedback subsystem.^23^ According to the computerized neural model of speech production and perception by Kröger, Kannampuzha and Neuschaefer-Rube (2009),^24^ the verbal praxic difficulties are encountered in the motor plane, which defines the temporal coordination of speech gestures or vocal tract action units. Thus, upon the occurrence of emission errors committed by speakers with apraxia of speech, such as substitution, omission, addition, anticipation, reiteration and metathesis, disturbances in the motor plane stage occur and activation of feedback is necessary to enable self-correction to take place, as per the speech processing model of Guenther et al. (2006).^23^

Our finding that the addition error type was relatively uncommon is in line with the results of the studies by Odell et al. (1990) and McNeil et al. (1997).^14,21^ Conversely, Deal and Darley (1972) found the addition error type to be more frequently occurring than self-correction error types.^5^ Our results corroborate the findings of other studies involving phonological analysis of errors committed by aphasics.^25-27^ Two of these studies included Broca’s aphasics who presented apraxia of speech secondary to aphasia. Only one participant in our study did not present an aphasic picture associated with apraxia of speech, and therefore this type of emissive error in our casuistic may be more related to language alteration.

Concerning sequential errors (anticipation, reiteration and metathesis), LaPointe and Johns (1975) found anticipation errors to exceed reiteration errors.^12^ It is noteworthy that these authors deemed sequencing errors to be substitution-type errors, which is in fact the case. However, in our study we provided a breakdown of substitution which showed sequential errors in anticipation, reiteration and metathesis. Romani et al. (2002) studied phonological error types in aphasics and found the metathesis/transposition type error to be the least frequently occurring,^27^ a result replicated in our sample.

Hesitation and prolongation error frequencies were similar. Many studies have shown hesitation and prolongation to be common in apraxia pictures.^2,4,7,9-11,16,20^ Kent and Rosenbek (1983) showed a variety of different segmental and prosodic abnormalities in the speech of individuals with apraxia and aphasia, including slow speaking rate with prolonged transitions, steady states, inter-syllable pauses, initiation difficulties, and errors of selection or sequencing of segments.^11^

Based on the results found we can confirm that, in terms of frequency of the error types studied, omission was one of the most commonly occurring. This finding differs to results described in some other studies assessing the speech of patients with apraxia. Furthermore, the addition error presented the lowest mean of the error types studied, a finding previously observed only in studies involving speakers with aphasia associated with apraxia.

Riecker et al. (2008)^28^ revealed a significant effect of syllable onset complexity on speech motor control yet a significant effect of syllable frequency was not evident. Structural differences between the Portuguese and English languages lead to differences in syllable onset complexity and its effect on motor control. This feature may contribute to differences in the pattern of errors in the languages.

Thus, the present study characterized error types and frequencies in the speech of patients with apraxia of speech, complementing the study by Cera and Ortiz (2009) in speakers of Portuguese with apraxia^3^ in which the most frequently substituted and omitted phonemes, along with the profile of these substitutions, were analyzed.

This study should be considered in the light of limitations, namely that the small sample size precludes generalization of findings.
